# High Thermoelectric Power Generation by SWCNT/PPy Core Shell Nanocomposites

**DOI:** 10.3390/nano12152582

**Published:** 2022-07-27

**Authors:** M. Almasoudi, Numan Salah, Ahmed Alshahrie, Abdu Saeed, Mutabe Aljaghtham, M. Sh. Zoromba, M. H. Abdel-Aziz, Kunihito Koumoto

**Affiliations:** 1 Department of Physics, Faculty of Science, King Abdulaziz University, Jeddah 21589, Saudi Arabia; malmasoudi0020@stu.kau.edu.sa (M.A.); aalshahri@kau.edu.sa (A.A.); abdusaeed79@hotmail.com (A.S.); 2 Department of Physics, Al-Qunfudah University College, Umm Al-Qura University, Makkah 21955, Saudi Arabia; 3 K. A. CARE Energy Research and Innovation Center, King Abdulaziz University, Jeddah 21589, Saudi Arabia; 4 Center of Nanotechnology, King Abdulaziz University, Jeddah 21589, Saudi Arabia; g44233a@cc.nagoya-u.ac.jp; 5 Department of Mechanical Engineering, College of Engineering, Prince Sattam bin Abdulaziz University, Al Kharj 16273, Saudi Arabia; m.aljaghtham@psau.edu.sa; 6 Department of Chemical and Materials Engineering, King Abdulaziz University, Rabigh 21911, Saudi Arabia; mzoromba@kau.edu.sa (M.S.Z.); mhmossa@kau.edu.sa (M.H.A.-A.); 7 Department of Chemistry, Faculty of Science, Port Said University, Port-Said 42521, Egypt; 8 Department of Chemical Engineering, Faculty of Engineering, Alexandria University, Alexandria 5424041, Egypt; 9 Nagoya Industrial Science Research Institute, Nagoya 464-0819, Japan

**Keywords:** thermoelectric materials, conducting polymers, polypyrrole, single-wall carbon nanotubes, core shell nanocomposites

## Abstract

Polypyrrole (PPy) is a conducting polymer with attractive thermoelectric (TE) properties. It is simple to fabricate and modify its morphology for enhanced electrical conductivity. However, such improvement is still limited to considerably enhancing TE performance. In this case, a single-wall carbon nanotube (SWCNT), which has ultrathin diameters and exhibits semi-metallic electrical conductivity, might be a proper candidate to be combined with PPy as a core shell one-dimensional (1D) nanocomposite for higher TE power generation. In this work, core shell nanocomposites based on SWCNT/PPy were fabricated. Various amounts of pyrrole (Py), which are monomer sources for PPy, were coated on SWCNT, along with methyl orange (MO) as a surfactant and ferric chloride as an initiator. The optimum value of Py for maximum TE performance was determined. The results showed that the SWCNT acted as a core template to direct the self-assembly of PPy and also to further enhance TE performance. The TE power factor, *PF*, and figure of merit, zT, values of the pure PPy were initially recorded as ~1 µW/mK^2^ and 0.0011, respectively. These values were greatly increased to 360 µW/mK^2^ and 0.09 for the optimized core shell nanocomposite sample. The TE power generation characteristics of the fabricated single-leg module of the optimized sample were also investigated and confirmed these findings. This enhancement was attributed to the uniform coating and good interaction between PPy polymer chains and walls of the SWCNT through π–π stacking. The significant enhancement in the TE performance of SWCNT/PPy nanocomposite is found to be superior compared to those reported in similar composites, which indicates that this nanocomposite is a suitable and scalable TE material for TE power generation.

## 1. Introduction

In recent years, there has been rapid development in organic thermoelectric materials, especially conducting polymers and their composites due to several advantages over inorganic thermoelectric materials. These conducting polymers exhibit light weight, lower cost, reasonable thermal conductivity, easy fabrication process, and excellent flexibility. For instance, the thermoelectric performance of organic polymers can be significantly improved by controlling the combination of carbon nanotubes (CNTs) or graphene nanosheets with such polymers [1,2,3]. So far, the most common conducting polymers that have been investigated as thermoelectric materials are polyaniline (PANI) [4], polypyrrole (PPy) [5], and poly (3,4-ethylene dioxythiophene) (PEDOT) [6,7,8]. However, the TE performance was limited because of the low electrical conductivity of the used carbon nanostructures or due to the low interaction between the conducting polymer and carbon material [9,10]. Tuning the morphology of such polymers was also performed in [6,11,12]; however, no significant improvement in the TE performance was reported.

The conducted work on PPy-based composites as a TE material is less compared to that reported on PEDOT and PANI composites, but recent studies showed that when PPy is doped with appropriate dopants or included in a proper composite material, it shows good mechanical properties, high electrical conductivity, and low thermal conductivity, which therefore enhance the TE performance [13]. The nanostructure form of PPy was also reported to have a considerable TE performance [9,14,15,16], especially the PPy nanotube, which showed remarkable improvement in TE performance [13]. Multiwall carbon nanotubes (MWCNT) or single-wall carbon nanotubes (SWCNT) were also used to enhance the TE properties of PPy [10,17,18,19]. The effect of other carbon nanostructures such as graphene nanosheets [20] or reduced graphene oxide (rGO) [21] was also investigated to obtain the TE performance of the PPy. However, it is still necessary to explore other approaches or suitable precursors that can be developed, such as a one-dimensional (1D) core shell structure of CNTs/PPy, with a smooth coating. This coating might facilitate charge transport and thus increases the electrical conductivity. It also can generate extra energy filtering sites at the interfaces between the PPy and CNTs.

The recent work reported in our lab on PPy [22,23] and PPy with carbon nanotubes [20,22] was focused on enhancing the TE performance of this polymer, but these efforts were focused either on the effect of surfactant type [22] or the surfactant [23] and carbon nanotubes [18] concentrations on the TE performance of the PPy. Although these studies demonstrate the capability of this polymer as TE, the observed TE performance is still low for real application as TE materials. One of the most important factors to enhance the TE performance of PPy is the selection of highly conducting carbon nanotubes such as single-wall carbon nanotubes, which have not been well addressed. It is understood that the electrical conductivity of carbon nanotubes can vary from semiconductors to metallic. This TE property depends on several factors such as diameters and chirality, and even the SWCNT, which is considered the best interim of their electrical conductivity, can vary from low semi-conductive to metallics [24]. In this case, coating PPy with highly conducting SWCNT along with an electrical conductivity value larger than 50,000 S/m (in a pressed compact form) might be quite important to enhance the TE performance. Moreover, selecting the proper surfactant and oxidant are also important for developing a 1D core shell structure with a smooth coating.

In this work, PPy was synthesized in the presence of methyl orange (MO) as a surfactant to regulate its shape as a blank thermoelectric polymer. Subsequently, highly conducting SWCNTs along with various concentrations of PPy were produced in the form of 1D core shell nanocomposite structures (SWCNT/Ppy) using an in situ polymerization method. The manufactured SWCNT/PPy nanocomposites were characterized by several well-known techniques such as SEM, TEM, Raman, FTIR, and XPS spectroscopy to analyze the TE performance. Moreover, the power generation characteristics of a single-leg module of the optimized SWCNT/PPy core shell nanocomposite were numerically and experimentally quantified. Additionally, the TE performance of the present SWCNT/PPy nanocomposite is found to be superior compared to previous studies reported in the literature.

## 2. Materials and Methods

### 2.1. Materials

Highly pure pyrrole monomer (Py), ethanol, anhydrous ferric chloride (FeCl_3_.6H_2_O), and methyl orange (MO) were purchased from Sigma-Aldrich, Steinheim, Germany. All reagents were of analytical grade (99.99%) and used as received without further purification. Single-wall carbon nanotubes (SWCNTs) of high electrical conductivity (>1000 S/cm in their pellet form) were purchased from Ad-Nano Technologies (Karnataka, India).

### 2.2. Synthesis of SWCNT/PPy Nanocomposites

Pure PPy was prepared by dissolving 750 mg of MO and 1 mL of Py monomer in 50 mL of absolute ethanol, and the resulting solution was diluted by DI water up to 200 mL using a magnetic stirrer (800 rpm) at room temperature (RT) for 20 min. In another beaker, 2340 mg of FeCl_3_.6H_2_O as an oxidant agent was dissolved in 200 mL of DI water, which was then added dropwise to the Py solution. The mixture was magnetically stirred for 48 h. The resulting black precipitation was filtered and washed several times successively with DI water and absolute ethanol to remove the surfactant and unreacted species. Then, the resulting product was dried at 60 °C for 24 h. The SWCNT/PPy nanocomposites were prepared as follows. A typical amount of SWCNT (300 mg) was dispersed in 50 mL of ethanol; then, this solution was diluted by 150 mL of DI water and sonicated for 3 h. The desired amounts of MO and Py monomer were added to the previous solution under continuous stirring for 30 min. Then, under vigorous stirring, the corresponding amount of FeCl_3_.6H_2_O in 200 mL of DI water was added slowly dropwise into the above solution to initiate the polymerization reaction and the mixture was magnetically stirred for two days. The product was filtered, washed, and dried exactly as in the case of pure PPy. Table 1 shows SWCNT amounts and the chemical materials used for this coating.

### 2.3. Characterizations

The morphology and microstructure of the pristine SWCNT, the neat PPy, and SWCNT/PPy nanocomposites were investigated using scanning electron microscopy (SEM) (JSM-7500F, JEOL, Tokyo, Japan) and transmission electron microscopy (TEM) (JEM 2100F, JEOL). Raman spectra were acquired using micro-Raman spectroscopy (Thermo Fisher Scientific, Waltham, MA, USA), whereas the FTIR spectra of the samples were derived by attenuated total reflection–Fourier transform infrared (ATR-FTIR) spectrometry (Thermo Fisher Scientific, Waltham, MA, USA). X-ray diffraction (XRD) studies were carried out using an Ultima-IV X-ray diffractometer (Rigaku, Japan) equipped with Cu Kα radiation (k = 1.5406 Å), while the surface composition changes of the synthesized samples were examined using X-ray photoelectron spectroscopy (XPS, PHI 5000 VersaProbe, Japan). To investigate the thermoelectrical properties, the pellets of the pure and SWCNT/PPy nanocomposites were prepared under a pressure of 15 tons utilizing a manual hydraulic press. The prepared pellets, which have dimensions of 13 mm diameter and 1.5–2.0 mm thickness, were annealed at 370 K for one hour in an oven under a vacuum atmosphere, and then their thermoelectric properties were measured.

The electrical resistivity and Seebeck coefficient measurements from 300 to 370 K were accomplished using the LSR-3 (Linseis GmbH) in the He atmosphere. The heating rate was fixed at 5 K/min and the temperature gradient between the cold and hot sides was set at 50 K. Thermal conductivity of the fabricated pellets was then determined using the laser flash method in LFA-1000 (Linseis, Selb, Germany). The measurements were performed perpendicular to the surface of the pressed pellet in a vacuum atmosphere and the pellet sides’ heating rate was set at 10 K/min. The charge carrier density and Hall mobility of prepared samples at RT were determined using the HCS 10 system, Linseis. To measure the output power for pure SWCNT, PPy, and SWCNT/PPy nanocomposites, rectangular (4 × 6 × 10 mm) single-leg modules were fabricated using a manual hydraulic press. These modules were fixed on a ceramic substrate, and both sides of the modules were connected to the measuring apparatus using aluminum electrodes. Strips of aluminum electrodes were used to cover the two sides. One side of the modules was placed on the hot plate that was heated to the maximum temperature (370 K). To measure the output voltage and current, a high sensitivity I–V measurement system (Keithley Instruments, Solon, OH, USA) was utilized.

## 3. Results and Discussion

**Figure 1:**
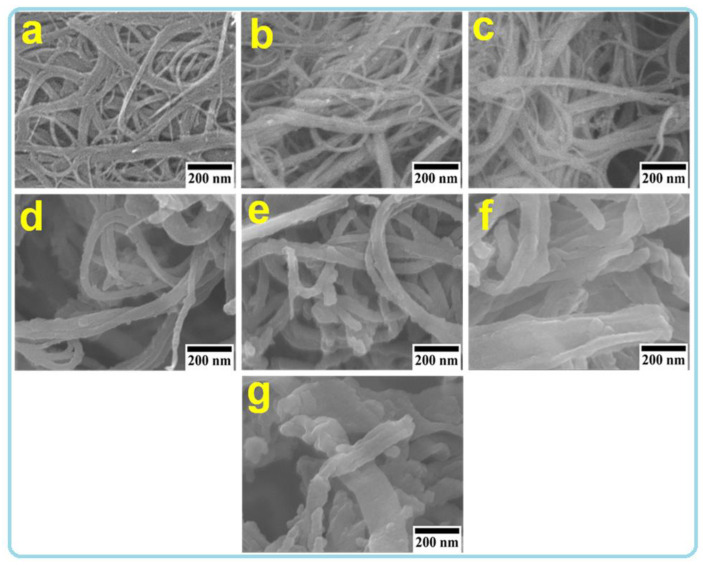
SEM images at the same magnification of SWCNT/PPyl nanocomposites. Images of pure SWCNT and PPy are also shown. (**a**) SWCNT, (**b**) PW1, (**c**) PW2, (**d**) PW3, (**e**) PW4, (**f**) PW5, and (**g**) PPy.

**Figure 2:**
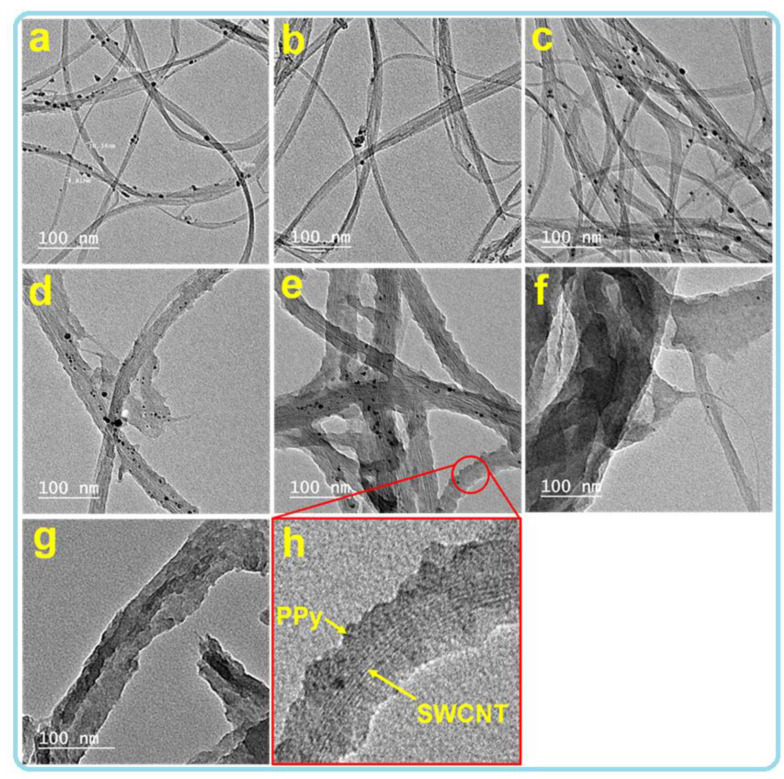
TEM images obtained at the same magnification for SWCNT/PPy nanocomposites. Images of pure SWCNT and PPy are also shown. (**a**) SWCNT, (**b**) PW1, (**c**) PW2, (**d**) PW3, (**e**) PW4, (**f**) PW5, (**g**) PPy. The image in (**h**) is a high-resolution TEM image for the PW4 sample.

The surface morphology and microstructure of the produced SWCNT/PPy nanocomposites were investigated by both SEM and TEM techniques, as shown in Figure 1; Figure 2. The SEM images show that the PPy was clearly formed on the sidewalls of the SWCNT, resulting in a one-dimensional (1D) nanocomposite (Figure 1). SEM images reveal that the PPy smooth coating had a considerable impact on the diameter of the SWCNT. As a systematic coating could be seen, smaller amounts of PPy could result in thinner shells/layers of PPy on the walls of SWCNT, while higher amounts of PPy could produce thicker layers of PPy. This coating was achieved by using different amounts of PPy (increasing the amount of PPy from 0.05 to 2 mL) and fixing the amount of SWCNTs, as displayed in Table 1. As shown in Figure 1a, the SWCNTs exist mostly in bundles with various diameters, and therefore, the formed core shell nanocomposites are present in various diameters (Figure 1b–f), which can be also seen clearly in the TEM images in Figure 2. From this figure, the uncoated and coated PPy-SWCNT with different PPy layer thicknesses show a similar trend to that observed in SEM images. As the amounts of PPy increase, the coating thickness increases, as shown in Figure 2a–g. Moreover, it can be clearly seen the SWCNT is situated in the cores of these 1D structures, whereas the PPy forms the shells (Figure 2h). This kind of smooth coating was also observed previously in MWCNT/PPy [25].

Raman and FTIR spectra of the SWCNT, PPy, and the SWCNT/PPy core shell nanocomposites were recorded and are presented in detail in the supplementary data in Figures S1 and S2, respectively, while the results of utilizing the XRD pattern of these samples are presented in Figure S3. The elemental composition and chemical states of C1s present in the SWCNT, PPy, and SWCNT/PPy core shell nanocomposites were investigated using the XPS technique, as demonstrated in Figures S4 and S5. The XPS survey profiles of these samples are shown in Figure S4, while the band of C1s of the uncoated SWCNT, SWCNT/PPy nanocomposites, and pure PPy was deconvoluted and presented in Figure S5a–g. The results are described and interpreted in the Supplementary Materials.

**Figure 3:**
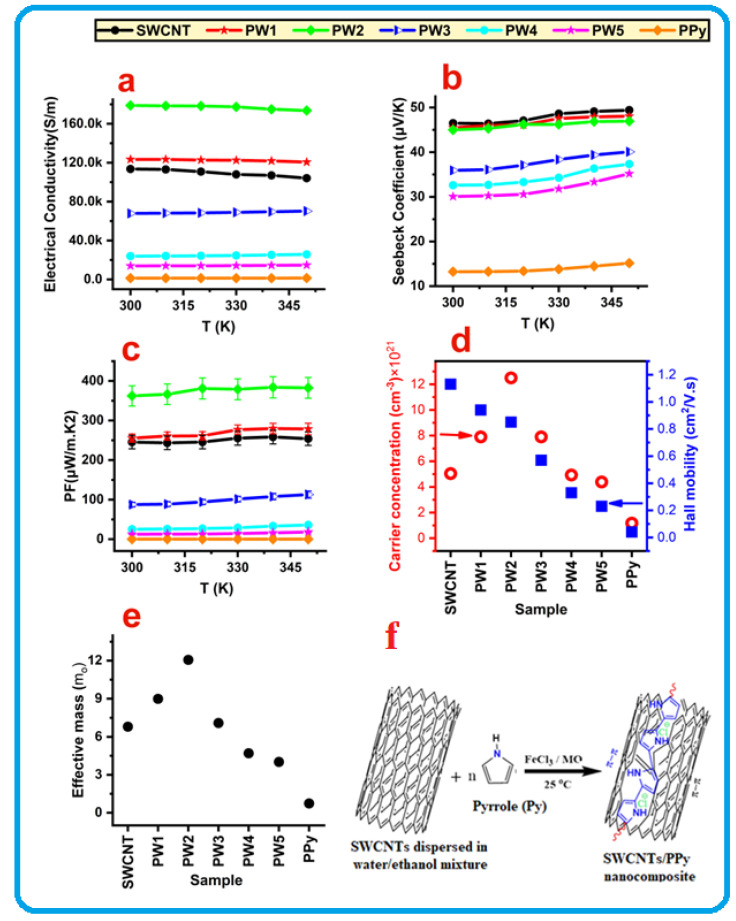
TE performance of the SWCNT/PPy nanocomposites as a function of the temperature (**a**–**c**). The charge carrier concentration, Hall mobility (**d**), effective mass (**e**) at RT of the pure PPy, SWCNT, and SWCNT/PPy nanocomposite samples are also shown. (**f**) is a schematic illustration showing the π–π conjugation interaction between Ppy and SWCNT during the polymerization.

The TE properties of the pure PPy, SWCNT, and SWCNT/PPy core shell nanocomposites (PW1–PW5) are presented in Figure 3a–e. At room temperature (RT), the measured electrical conductivity for pure PPy and SWCNT is approximately equal to 1330 and 113,510 S/m, respectively (Figure 3a). When the temperature increases to 350 K, the value of PPy slightly rises to 1455 S/m, while the SWCNT decreases to 104,069 S/m. PPy showed semiconductor behavior while SWCNT showed typical metallic conducting behavior. Therefore, the electrical conductivity of the produced PPy molecules is comparable to or even higher than those with similar structures or morphologies, as reported in the literature [20,35]. The electrical conductivity of the SWCNT/PPy nanocomposites with different PPy layer thicknesses is presented in Figure 3a. The obtained values were recorded as a function of temperature in the temperature range of 300–350 K. The values of coated SWCNT at RT were increased with an increase in the thickness of the coating PPy layer, mainly of the PW1and PW2 samples. The measured values at RT are equal to 123,000 and 178,000 S/m for PW1 and PW2, respectively. These values were decreased with an increase in the temperature up to 350 K, reaching 120,616 S/m for the PW1 sample and 173,637 S/m for PW2 samples, indicating degenerate semiconductor behavior. In contrast, the electrical conductivity values of the remaining samples (PW3, PW, and PW5) were decreased with the increase in thickness of the coating PPy layer. At RT, the electrical conductivity of PW3, PW4, and PW5 is approximately 68,000, 24,000, and 14,000 S/m, respectively, while at 350 K, the electrical conductivity values of these samples (PW3, PW4, and PW5) were 25–30% higher than those recorded at RT, which explains the semiconductor behavior.

This substantial improvement in the electrical conductivity of the SWCNT by Ppy coating at optimum concentration is remarkable, which is probably attributed to the smooth and flawless Ppy coating generated on the wall of SWCNT. This coating perhaps assisted charge transport and boosted the charge concentrations, simply by combining the carriers that exist in both the SWCNTs and Ppy. These findings match those of Ppy nanowire/graphene nanocomposites reported previously [35]. Furthermore, according to previous studies reported in the literature [36], the electrical conductivity of SWCNT/Ppy core/shell nanocomposites may be improved by using SWCNT as a template for the self-assembly of Ppy. This, therefore, enhances the order of crystalline alignments by the strong π–π conjugation interaction between Ppy and SWCNT during the polymerization. This interaction is shown in Figure 3f.

The measured Seebeck coefficients, as a function of temperature for the pure PPy, SWCNT, and SWCNT/PPy nanocomposites (PW1–PW5), are displayed in Figure 3b. All tested samples had a positive Seebeck coefficient, indicating a p-type conductive behavior. At 300 K, the measured Seebeck coefficients of SWCNT and PPy are equal to 46.5 μV/K and 13.2 μV/K, respectively; however, these values were slightly increased by heating the samples to 350 K. The nanocomposite samples (PW1–PW5) showed intermediate values between those of SWCNT and PPy. However, the samples of PW1 and PW2 have closer Seebeck values to that of SWCNT. These remarkable results for TE application were mainly obtained for PW2, which was also found to have the highest electrical conductivity. The thicker layers of PPy on SWCNT for PW3–PW5 significantly reduced the Seebeck values, but they were still higher than the Seebeck values of PPy.

The calculated power factor (PF) values of the pure PPy, SWCNT, and SWCNT/PPy nanocomposites were plotted as a function of temperature, and the obtained results are shown in Figure 3c. At RT, the power factor values of the uncoated SWCNT and PPy were found to be around 245 and 0.2 µW/mK^2^, respectively. By excluding samples of PW1 and PW2, the calculated PF of the SWCNT/PPy nanocomposites decreases for the thicker coating layer of the PPy. The PF value of the PW2 sample is equal to 362 µW/mK^2^, which is higher than values of the pure or other nanocomposite samples. This value is achieved due to the enhancement in the electrical conductivity of the PW2 sample (Figure 3a). This is likely due to the use of well-coated PPy (that have fewer defects or agglomerations) on the surface of the selected highly conducting SWCNT (Figure 2; Figure 3). The other coated samples with thicker layers of PPy (PW3–PW5) seem to contain some defects and agglomerations of PPy and also some residuals of MO molecules on the sidewalls of the SWCNT (see Figure S1). This is expected to reduce the charge transport and decrease the electrical conductivity. In general, the use of SWCNT as a template for the self-assembly of PPy will facilitate the crystalline alignments of PPy chains and make them more ordered by the strong π–π conjugation interaction between PPy and SWCNT during polymerization. This interaction will enhance the electrical conductivity by acquiring more carriers, as PPy is rich with charge carriers (see Figure 3d), but at certain layer thickness of highly pure PPy (with no defects/agglomerations or residuals). It is worth mentioning that the recorded PF value of the optimum nanocomposite sample in this study, PW2, is greater than the PF values of MWCNT/PPy and SWCNTs/PPy composites reported in the literature [36,37].

Hall effect characterization of the PPy, SWCNT, and SWCNT/PPy nanocomposite samples was performed to demonstrate the effect of PPy coating on the SWCNT in terms of electrical conductivity. Figure 3d depicts the charge carrier concentration and Hall mobility of these samples at 300 K. From the figure, the maximum carrier concentration of 12.5 × 10^21^ cm^–3^ was achieved for the PW2 (SWCNT/PPy (0.1)), whereas the corresponding Hall mobility was equal to 0.85 cm^2^/Vs. On the other hand, the pure PPy recorded the lower charge carrier concentration of 1.1 × 10^21^ cm^–3^, while the Hall mobility was around 0.05 cm^2^/Vs. Moreover, the carrier concentration and the Hall mobility of SWCNT are approximately equal to 5 × 10^21^ cm^–3^ and 1.1 cm^2^/Vs, respectively. The charge carrier concentration was increased dramatically due to the coated SWCNT with a thinner PPy layer, especially in the PW2 sample. This indicates that the carrier concentration is a crucial factor to enhance electrical conductivity by PPy coating on the SWCNT walls. The electrical conductivity value of the coated sample of PW2 was 57% higher than that value of SWCNT (Figure 3a). In general, it is noticeable that coating SWCNTs with PPy is a useful approach to enhance the electrical conductivity and similarly might be suitable for other carbon nanotubes (CNTs) [18,25].

To further elaborate on the electrical conductivity, the effective mass (m*) values were also estimated, as displayed in Figure 3e. The above results of the electrical conductivity (Figure 3a) show that the core shell nanocomposites seem to be degenerate semiconductors, mainly for PW1 and PW2 samples; therefore, the effective mass m* can be obtained from the Seebeck coefficient, S, and the carrier concentration, *n* (*p* for holes), according to Pisarenko’s relationship [38]:

(1) $$S=\frac{8\pi^{2}k_{B}{}^{2}}{3eh^{2}}m^{*}T{\left(\frac{\pi }{3n}\right)}^{2/3}$$

where *k_B_* is the Boltzmann constant = 1.38 × 10^−23^ m^2^kgs^−2^K^−1^; *h* is Planck’s constant = 6.63 × 10^−34^ m^2^kg/s; and *e* = the electron charge = 1.6 × 10^−19^ Coulombs.

Figure 3e shows that the maximum effective mass value is equal to around 12 m_o_ for the PW2 (SWCNT/PPy (0.1)). The high effective mass value along with the relatively high value of mean free bath for carrier scattering might be the reasons for the electrical conductivity enhancement in this sample (PW2). This was perhaps facilitated due to the smooth coating of PPy on the SWCNT sidewalls, which enables the charge transport. On the other side, the pure PPy showed the lowest effective mass of 0.7 m_o_, while the mean free path was equal to around 0.01 nm. Moreover, the effective mass of neat SWCNT was around 7 m_o_. In the case of thicker PPy coatings (PW3–PW5), the effective mass values were decreased with increasing the thickness of the PPy layer. This perhaps is due to the presence of some branches or agglomerations of PPy particles within the coated layers ( Figure 1; Figure 2).

**Figure 4:**
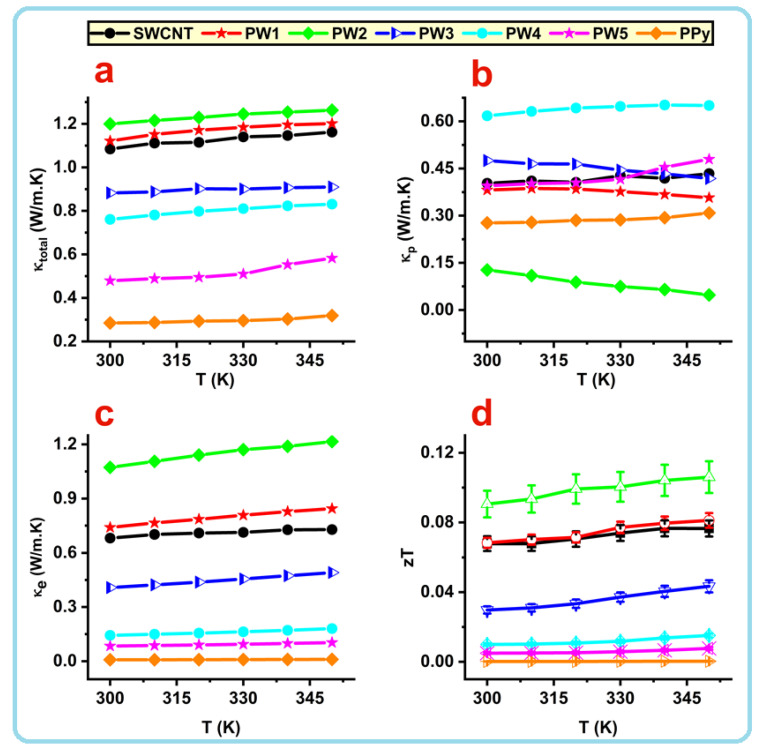
Total thermal conductivity, $\kappa_{total\;}$(**a**), phonon thermal conductivity, $\kappa_{p}$ (**b**), electron thermal conductivity, $\kappa_{e}$ (**c**), and figure of merit, zT (**d**) of the SWCNT/PPy core shell nanocomposites as a function of temperature.

The thermal conductivity ($\kappa_{\mathrm{total}}$) of a material can be described as the sum of electronic thermal conductivity and phonon’s lattice thermal conductivity. This means that thermal conductivity is the total amount of heat transferred through the material by electron/hole transporting ($\kappa_{e}$) and that which is transferred by phonons traveling ($\kappa_{p}$). The value of $\kappa_{e}$ can be obtained using the measured electrical conductivity and then the Wiedemann–Franz law [39] on the premise that the relaxation times of phonons and electron holes are the same.

(2) $$L=\frac{\kappa_{e}}{\sigma T}=\frac{\pi^{2}k_{B}^{2}}{3e^{2}}=2\times 10^{-8}{W\Omega K}^{-2}$$

where *L* is the Lorenz number, $k_{B}$ is Boltzmann’s constant, and *e* is the electron charge. The values of $\kappa_{\mathrm{total}}$, $\kappa_{p},$ and $\kappa_{e}$ of the neat PPy, SWCNT, and SWCNT/PPy core shell nanocomposites (PW1–PW5) are presented as a function of temperature in Figure 4a–c.

At RT, the $\kappa_{\mathrm{total}}$ of SWCNT was equal to 1.08 W/mK. When the temperature was raised to 350 K, this value slightly increased to around 1.16 W/mK (Figure 4a). These values are low compared to those published in the literature [40]. This may be due to the presence of SWCNTs in bulk bundles with a complex network that has many interfaces (Figure 1a). The $\kappa_{\mathrm{total}}$ in the case of pure PPy is around 0.3 in the temperature range of room temperature to 350 K. This value is similar to those values reported in the previous study [23]. It is obvious that the $\kappa_{\mathrm{total}}$ of SWCNT appears to be almost temperature-dependent, which differs from the case of pure PPy sample. As the temperature increases, network structures may expand, resulting in close contact between nearby nanotubes of SWCNT. Consequently, it may minimize phonon scattering sites and enhance phonon transit, which therefore leads to increased thermal conductivity, $\kappa_{\mathrm{total}}$.

The *κ*_*p*_ of SWCNT is small compared to its $\kappa_{\mathrm{total}}$, while the *κ*_*p*_ of pure PPy is closer to its $\kappa_{\mathrm{total}}$, as displayed in Figure 4a,b. This interestingly indicates that the electrons are the major heat carriers in the SWCNT sample, whereas the phonons are the major heat carriers in pure PPy. The $\kappa_{\mathrm{total}}$ of the SWCNT/PPy nanocomposite samples (PW1–PW5) are presented in Figure 4a. Those samples demonstrate a trend similar to that shown by electrical conductivity. Intentionally, $\kappa_{\mathrm{total}}$ increased with an increase in the wrapping layer of PPy on SWCNTs (PW1 and PW2). Then, it is decreased with an increase in the thickness of the coated layers, as can be seen in samples PW3–PW5. At RT, PW1 and PW2 samples recorded the highest thermal conductivity values of around 1.12 and 1.2 W/mK, respectively. These values slightly increased with an increase in temperature up to 350 K, reaching to 1.2 and 1.26 W/mK for samples PW1 and PW2, respectively. The $\kappa_{\mathrm{total}}$ of PW3, PW4, and PW5 samples were found to be approximately 0.9, 0.8, and 0.5 W/mK, respectively, at both RT as well as at high temperature (383 K). The $\kappa_{\mathrm{total}}$ of these samples (PW3–PW5) are lesser than those of uncoated SWCNTs. The effect of the interfacial sites between the CNTs and the PPy layer may play an important role in the scattering of more phonons. Normally, at high temperatures, the total thermal conductivity decreases due to the enhanced phonon scattering (role of *κ*_*p*_), but in the present case, the observed slight increases by heating might be due to the role of $\kappa_{e}$, which are found to be the major heat carriers or major contributors to the total thermal conductivity in most of these samples.

It is clear that PW2 has the lowest value of $\kappa_{p}$ (around 0.12 W/mK) but has the highest value of $\kappa_{e}$ (1.07 W/mK). This indicates that electrons are the major heat carriers in this 1D nanocomposite sample. The results in Figure 3a,d show that this sample possesses the highest electrical conductivity and the maximum charge carrier concentration. The PW3 sample shows that the contributions of $\kappa_{p}$ and $\kappa_{e}$ are almost equal, while the $\kappa_{p}$ of the remaining samples (PW4 and PW5) are closer to those of $\kappa_{\mathrm{total}}$. It is clear that the $\kappa_{e}$ contribution seems to be small in these nanocomposites, mainly in PW4 and PW5 samples. Nevertheless, the thermal conductivity of the SWCNTs coated with the optimum amount of PPy in this work is significantly lower than the values of similar composites described in the literature [20].

The assessments of figure of merit (zT) as a function of temperature for all prepared samples are displayed in Figure 4d. The zT was dramatically raised thanks to the flawless 1D coating of PPy on the sidewalls of the SWCNTs. The zT for these samples demonstrated a trend similar to that shown by electrical conductivity. At RT, the uncoated SWCNT value was equal to 0.067, while it slightly increased to 0.076 by heating to 350 K. The pure PPy exhibited significantly lower zT values than those of SWCN, with values equal to 0.5 × 10^−3^ at RT and 2.0 × 10^−3^ at 440 K. The zT values of the slightly coated SWCNTs (PW1 and PW2) were significantly higher than those of both the SWCNT and PPy. The highest zT value of 0.09 was acquired for the PW2 sample (SWCNT/PPy(0.1)) at RT and this value was increased to nearly 0.11 at 350 K.

To compare the TE performance of the present SWCNT/PPy nanocomposites in this work with those of the similar composites, Table 2 summarizes the comparison values of the electrical conductivity, Seebeck coefficient, and power factor at 300 K for the nanocomposite materials based on carbon nanotubes and PPy. From the table, it is obvious that the present SWCNT/PPy nanocomposites have superior TE properties. The optimum SWCNT/PPy nanocomposite of PW2 shows the highest PF and zT values. Moreover, this study presents a clear strategy to wrap highly conducting SWCNTs with smooth PPy layers without PPy agglomeration to form 1D core shell nanocomposite with superior TE performance. These improvements in the TE performance of the PPy-coated SWCNT nanocomposites are due to the utilization of highly conducting SWCNTs and, more particularly, this massive increase because of coating with PPy. The Seebeck coefficient is also reasonable, beside the low thermal conductivity, leading to a nanocomposite with a high zT value.

The main reasons for improving the TE properties of the SWCNT/PPy nanocomposite might be further elaborated. The first reason is using the SWCNT, which has high electrical conductivity, beside its large surface area and aspect ratio that acted as conducting bridges or conducting networks linking the PPy conducting domains [44]. The second explanation is the enhancement of the electrical conductivity of SWCNT/PPy core/shell nanocomposites, which could be increased by using SWCNT as a template for the self-assembly of PPy, which makes crystalline alignments more ordered by the strong π–π conjugation interaction between PPy and SWCNT during polymerization (Figure 3f) [45]. The third reason is the energy filtering effect at the SWCNT/PPy interfaces, where adequate potential boundary barriers positively permitted the carriers with high energy to pass, which enhances (or maintain) the Seebeck coefficient [20]. It is understood that forming core shell nanocomposites is expected to enrich the interface sites. These sites are clearly shown in the HRTEM image presented in Figure 2h. However, in the present case, such filtering effects could only maintain the Seebeck values of the PW1 and PW2 samples. This is almost in agreement with the explanation given by Neophytou et al. [46]. Moreover, since SWCNT has a complex network and the interfaces between the SWCNT/PPy nanocomposites themselves are intricate, a significant influence on phonon scattering might result in low thermal conductivity.

The presented results in this work showed a remarkable finding: SWCNTs’ electrical conductivity may be considerably increased by properly wrapping them with a conducting polymer such as PPy to produce a 1D core shell nanocomposite. This might be due to the fact that conducting polymers such as PPy possess this feature by using appropriate precursors and suitable surfactants during the polarization process. It is therefore recommended to apply this approach to other highly conducting carbon materials and other conducting polymers. This straightforward procedure may serve to optimize the TE performance of TE-based polymer materials and it does not require a lengthy procedure or the use of intricate ternary composites. However, the procedure should be addressed very carefully, as sometimes this might lead to a negative result; Wang et al. [47] reported that a decrease in the electrical conductivity of other polymers might occur after adding carbon nanotubes, stating that “polymer/carbon nanotube composites exhibit poorer electrical conductivity than pure carbon nanotubes”.

**Figure 5:**
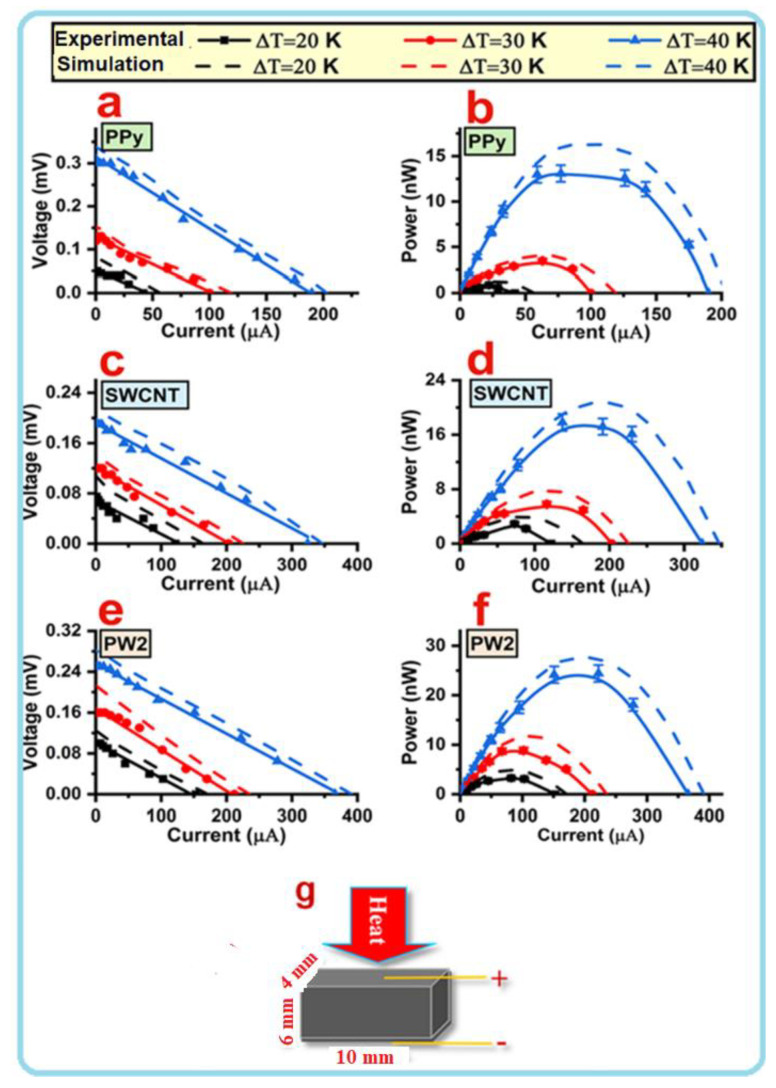
TE power generation characteristics of the single-leg module of the SWCNT/PPy core shell nanocomposite of sample PW2. The same for pure SWCNT and PPy are also shown for comparison. The simulated curves performed by ANSYS software are also shown (**a**–**f**). A single squared leg module is shown in (**g**).

A thermoelectric generator (TEG) is a small and solid-state apparatus employed to convert heat energy into electricity by a phenomenon called the thermoelectric Seebeck effect. In TEG devices, several factors should be examined, including the output voltage (V), current (I), and power (P) of the single-leg module generated by the temperature gradient between the two sides of a pressed compact. In real conditions, the power generation characteristics of the single cuboid-shaped leg modules composed of SWCNT, PPy, and SWCNT/PPy nanocomposite (PW2) were examined, and the results are given in Figure 5a–f. The measured V and P as a function of I under different ΔT showed that the SWCNT/PPy nanocomposite exhibited improved TE power compared to the individual samples. The dimensions of a single cuboid leg module are presented in Figure 5g. It can be seen that the V linearly decreases as the I increases at a fixed ΔT, and this I–V linear relationship remains unaltered when ΔT is changed; therefore, Ohm’s law holds true at any ΔT. Moreover, the V increased proportionally with the increase in ΔT due to the Seebeck effect. It has been also noted that the slopes of all I–V curves are nearly identical, demonstrating that ΔT has no major effect on the internal resistance (R) of all samples. As ΔT increases, the output power also increases; at ΔT = 40 K, the P_max_ of 24.42 nW (the equivalent power density is ~61 nW/cm^2^) was achieved for the PW2 sample. The experimental results were also validated by generating numerical simulation curves (Figure 5a–f). These curves were generated by a three-dimensional ANSYS numerical model with 20 node coupling elements, SOLID226, selected to simulate the thermoelectric current and voltage, similar to the work reported in the literature [48,49,50,51]. More details about this model are described in the Supplementary Data. From the figure, it can be concluded that the numerical simulation curves are in agreement with the experimental results.

The above findings show that the TEG of SWCNT/PPy(0.1) nanocomposites is superior compared to the individual samples, e.g., SWCNT and PPy. The attained findings are consistent with the PF and zT values of each leg, as shown in Figure 3c and Figure 4d. The PPy coating significantly improved the TE power output of the SWCNT and served as an excellent binder for the SWCNT, which is required for fabricating a strong leg. The results presented above concern only one leg of the SWCNT wrapped with a PPy semiconductor. Utilizing a large number of p–n pairs with acceptable matching might be used to create a functional electrical device with greater TE power generation.

## 4. Conclusions

In this work, a highly conducting SWCNT was combined with PPy as a 1D core shell nanocomposite for higher TE power generation. Various amounts of Py as a monomer source for PPy were coated on SWCNT along with methyl orange as a surfactant and ferric chloride as an initiator. The TE power factor and figure of merit values of the optimized core shell nanocomposite sample of SWCNT/PPy were greatly increased to 360 µW/mK^2^ and 0.09, respectively, which demonstrate significantly better TE performance than the individual materials. The power generation characteristics of a single-leg module of the optimized sample confirmed these enhancements. This enhancement was attributed to the uniform coating and good interaction between PPy polymer chains and walls of the SWCNT through π–π stacking. The achieved enhancement in the TE performance of SWCNT/PPy nanocomposite is found to be superior to those reported of similar composites based on carbon and PPy materials. It is therefore of importance to continue developing such nanocomposites that might be recommended as a scalable TE material for the future of generating high TE power.

## Figures and Tables

**Table 1:** Raw materials used for coating the SWCNT with PPy at a different layer thickness.

Samples	SWCNT (mg)	MO (mg)	Py (mL) (In 200 mL DI)	FeCl_3_ (mg) (In 200 mL DI)
PW1	300 mg	37.5	0.05	117
PW2		75	0.1	234
PW3		375	0.5	1170
PW4		750	1	2340
PW5		1500	2	4680

**Table 2:** The optimum thermoelectric properties of CNT/PPy composites.

Nanocomposite Materials	σ (S/m)	*S* (μV/K)	P.F. (μW/mK^2^)	zT	Ref.
PPy/rGO	4160	26.9	3.01	-	[21]
PPy/GNs (PPy/graphene nanosheets)	10,168	31.74	10.24	2.80 × 10^−3^	[20]
PPy/rGO thin film	8000	29	7.28	-	[41]
PPy nanowire/rGO	7500.1	33.8	8.56 ± 0.76	-	[12]
PPy/SWCNT (60 wt %)	39,900	22.2	19.7 ± 0.8	-	[19]
PPy/MWCNT (20 wt %)	~3150	~25.4	2.079		[36]
PPy/MWCNT (68 wt %) (at RT)	3670	24.5	2.2	-	[17]
PPy nanowire/SWCNT (60 wt %) (at RT)	~30,000	~25	21.7 ± 0.8	-	[10]
PPy/graphene (20 wt %) (at 380 K)	3690	16.6	1.01		[35]
SWCNT/PPy (40 wt %) (at 398 K)	10,699	22.59	5.46	-	[42]
PPy/SWCNTs film (at RT)	34,160	33.2	37.6 ± 2.3	-	[37]
MWCNTs/PPy PPy/acid-doped SWCNT	4000 385,000	~14 25	0.77 240.3	1 × 10^−3^ -	[18] [43]
					
**SWCNT/PPy (at RT)** **SWCNT/PPy (at 350 K)**	**178,835** **173,637**	**45** **47**	**362** **382**	**90 × 10^−3^** **110 × 10^−3^**	**This work**

## Data Availability

The data supporting the findings of this study are available upon request to nsalah@kau.edu.sa.

## Supplementary Materials

The following supporting information can be downloaded at: https://www.mdpi.com/article/10.3390/nano12152582/s1.
