# Supplementary material for: High Thermoelectric Power Generation by SWCNT/PPy Core Shell Nanocomposites
Source: Nanomaterials (Basel). 2022 Jul 27;12(15):2582. doi: 10.3390/nano12152582 (PMC9370189; doi:10.3390/nano12152582)
Supplement: Supplementary file 1 [file nanomaterials-12-02582-s001.zip › nanomaterials-1806828-supplementary.pdf]

nanomaterials

Supplementary Materials

High Thermoelectric Power Generation by SWCNT/PPy Core Shell Nanocomposites

M. Almasoudi [1,2], Numan Salah [3,4,*], Ahmed Alshahrie [1,4], Abdu Saeed [1], Mutabe Aljaghtham [5], M. Sh. Zoromba [6,7], M. H. Abdel-Aziz [6,8] and Kunihito Koumoto [4,9]

1 Department of Physics, Faculty of Science, King Abdulaziz University, Jeddah 21589, Saudi Arabia; malmasoudi0020@stu.kau.edu.sa (M.A.); aalshahri@kau.edu.sa (A.A.); abdusaeed79@hotmail.com (A.S.)
2 Department of Physics, Al-Qunfudah University College, Umm Al-Qura University, Makkah 21955, Saudi Arabia
3 K. A. CARE Energy Research and Innovation Center, King Abdulaziz University, Jeddah, 21589, Saudi Arabia
4 Center of Nanotechnology, King Abdulaziz University, Jeddah 21589, Saudi Arabia; g44233a@cc.nagoya-u.ac.jp
5 Department of Mechanical Engineering, College of Engineering, Prince Sattam bin Abdulaziz University, Al Kharj 16273, Saudi Arabia; m.aljaghtham@psau.edu.sa
6 Department of Chemical and Materials Engineering, King Abdulaziz University, Rabigh 21911, Saudi Arabia; mzoromba@kau.edu.sa (M.S.Z.); mhmossa@kau.edu.sa (M.H.A.-A.)
7 Department of Chemistry, Faculty of Science, Port Said University, Port-Said 42521, Egypt
8 Department of Chemical Engineering, Faculty of Engineering, Alexandria University, Alexandria 5424041, Egypt
9 Nagoya Industrial Science Research Institute, Nagoya 464-0819, Japan
* Correspondence: nsalah@kau.edu.sa or alnumany@yahoo.com

1. Raman spectra

Raman spectra were acquired as shown in Figure S1 to demonstrate the interfacial interaction between SWCNT and PPy coating layers. The spectra of uncoated SWCNT revealed two distinct intensity bands at 1344 and 1592 cm^{-1}, which are the result of disorder-induced vibration (D band) and graphite in-plane vibration (G band) bands, respectively [25]. The Pure PPy exhibited two strong bands at approximately 1390 and 1594 cm^{-1}, corresponding to the polymer backbone's C–C and C=C in-ring vibration modes, respectively [26,27]. In addition, the weak band at approximately 950 cm^{-1} might be assigned to the ring deformation associated with the di-cation (di-polaron) and the peak at 1080 cm^{-1} to the C–H in plane deformation [28]. The spectra of SWCNT/PPy nanocomposite are similar to that of pure PPy, but the range of amplitude decreases systematically by increasing the Py content. This reduction and band shift might imply that PPy and SWCNT have a very strong coupling and charge transfer. A considerable decrease in band intensities might have been achieved by a successful π–π stacking of SWCNT and PPy chains, particularly for the higher layer thickness of PPy [25]. Interestingly, it is clearly seen that several small bands were observed practically in pure PPy, PW3, PW4 and PW5 samples. To investigate the origin of those weak bands, the spectrum of the MO surfactant was also recorded, and the attained result is shown in the inset of Figure S1. This spectrum matches that reported value in the literature [29]. Hence, those weak bands in previously mentioned samples might be due to a residual MO on the sidewalls of the nanotubes.

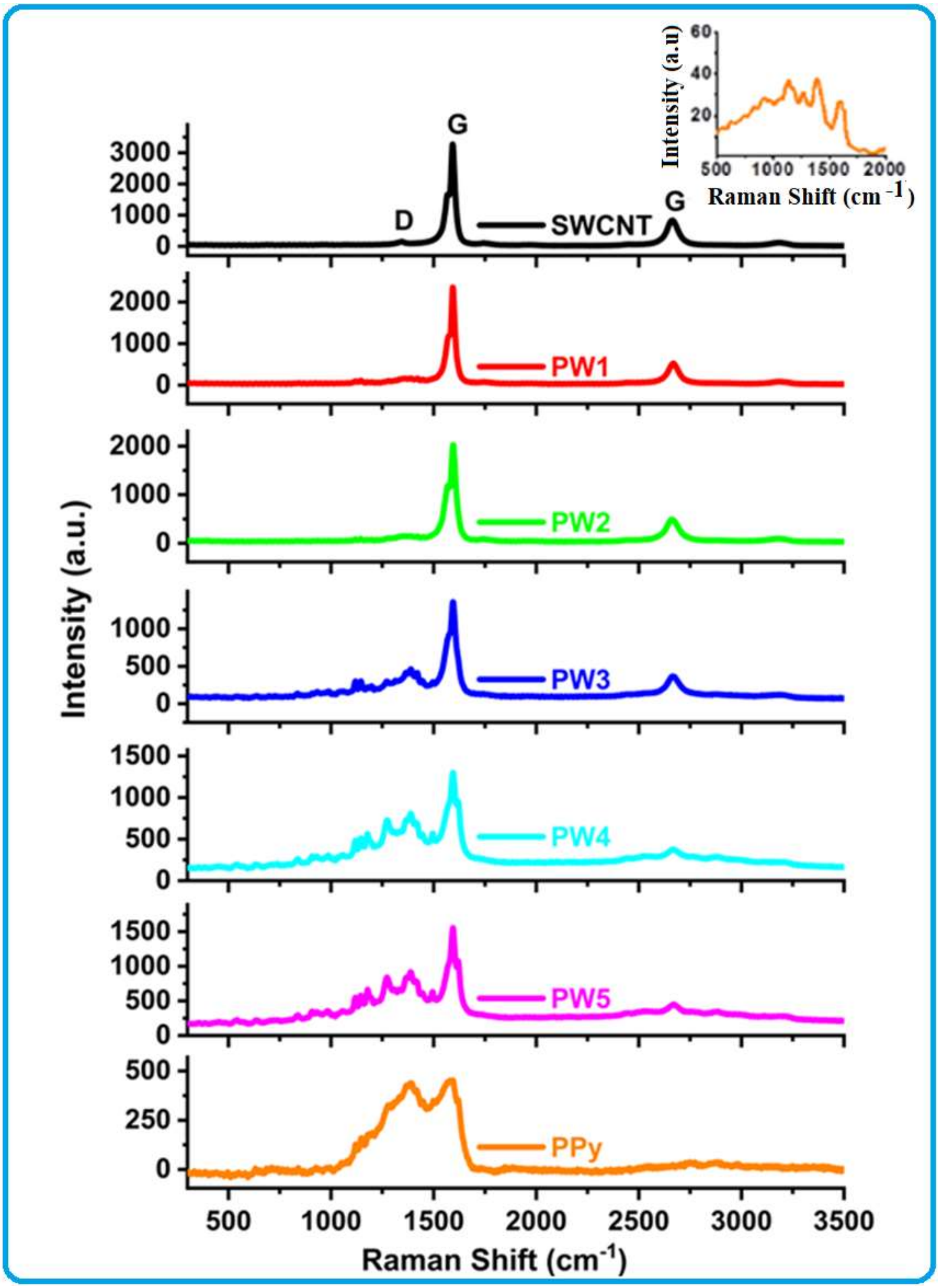

Figure S1. S. Raman spectra of the SWCNT/PPy core shell nanocomposites. Spectra of pure SWCNT and PPy are also shown. The inset displays the Raman spectrum of MO powder.

2. FTIR spectra

The FTIR spectra measurements of PPy and SWCNT/PPy core shell nanocomposites are shown in Figure S2. The spectrum of pure PPy shows several bands located at around 869, 964, 1025, 1157,1299,1452, 1540 and 1596 cm^{-1}. The first peak at 869 cm^{-1} might be attributed to C–H wagging, while the band at 964 cm^{-1} may be assigned to the C–C out-of-plane ring-deformation vibration [30]. The band located at 1025 cm^{-1} can be attributed to C–H in-plane deformation and N–H stretching vibration [31]. The C–N stretching vibration mode in the PPy is located at 1157 and 1452 cm^{-1}, [32] while the peak at 1299 cm^{-1} is due to C–H in-plane [33]. The peaks at 1540 and 1596 cm^{-1} are due to the C–C symmetric

ring vibration [32] and the C=C vibration mode of the polymer backbone [20], respectively. The FTIR spectra for SWCNT/PPy core shell nanocomposites are similar to those values for pure PPy and SWCNT. Notably, when SWCNT is added to the PPy matrix, PPy peaks shifted to the higher wave number line side. This shift may be due to the interactions, such as the π–π stacking between the PPy chains and SWCN. These results proved that PPy has been successfully formed in the side walls of SWCNT.

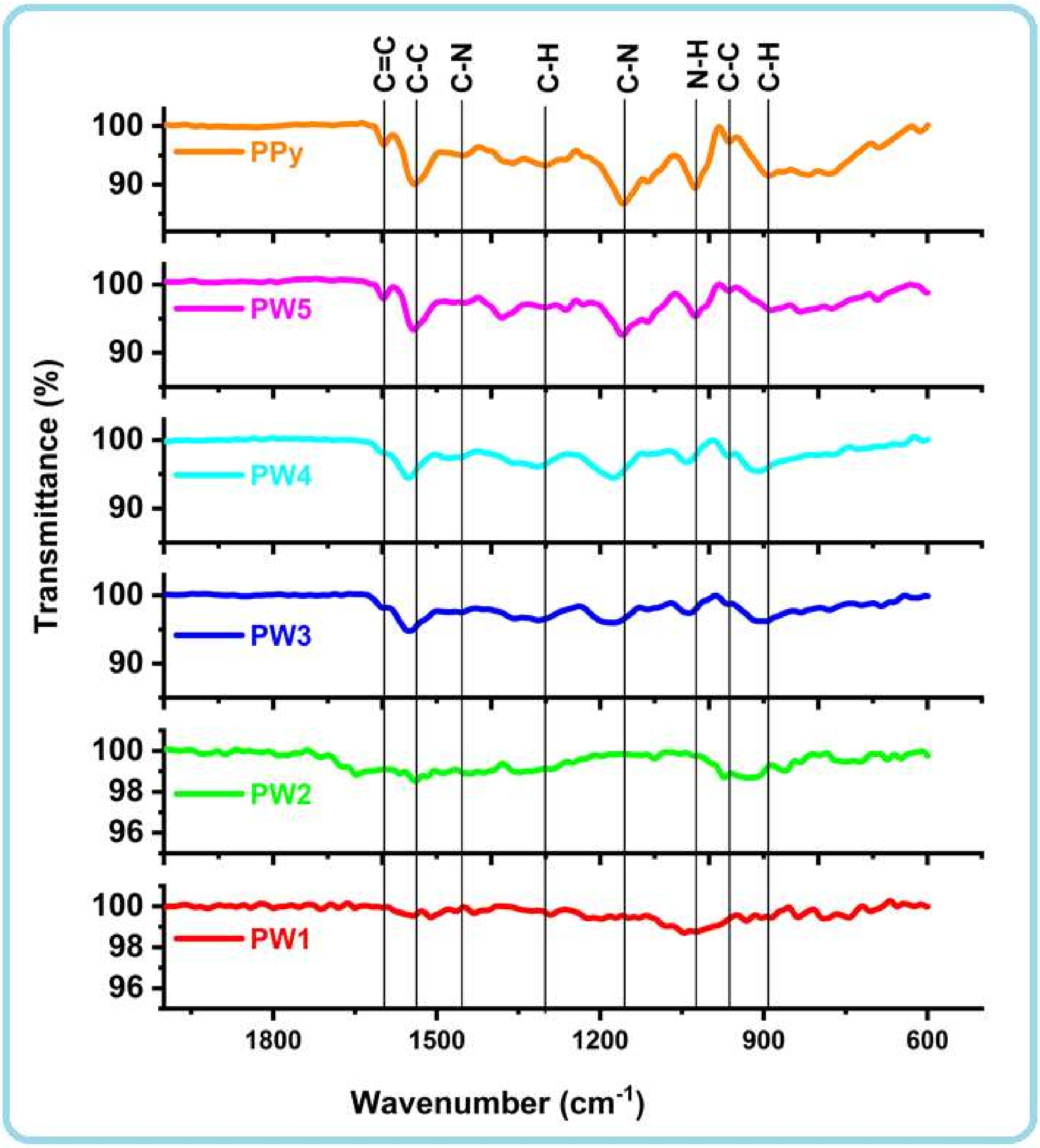

Figure S2. FTIR spectra obtained for SWCNT/PPy core shell nanocomposites. Spectra of pure SWCNT and PPy are also shown.

3. X-ray diffraction

Figure S3 represents the X-ray diffraction patterns for pure PPy and SWCNT/PPy core shell nanocomposites. The PPy pattern shows a broad peak at 2theta = 23.3° which signifies the characteristic peak of amorphous polypyrrole. For the pristine SWCNT, there are also two broad peaks at around 21° and 44°. As the amount of PPy increases in SWCNT/PPy composites, the diffraction peak at around 44° starts to decrease, while the peak at around 23° is still strong. This may prove that the PPy and SWCNT were entirely

interacted and the SWCNT were coated with a PPy layer. Similar observations were reported in the MWCNTs/ PPy composite [17] and the PPy/ graphene composite [20] Interestingly, PW5 and pure PPy samples show an extra sharp peak at 20° which might be because of the MO residue on the sidewalls of those samples as proven by the XRD pattern for MO powder (the inset in Figure S3) [23].

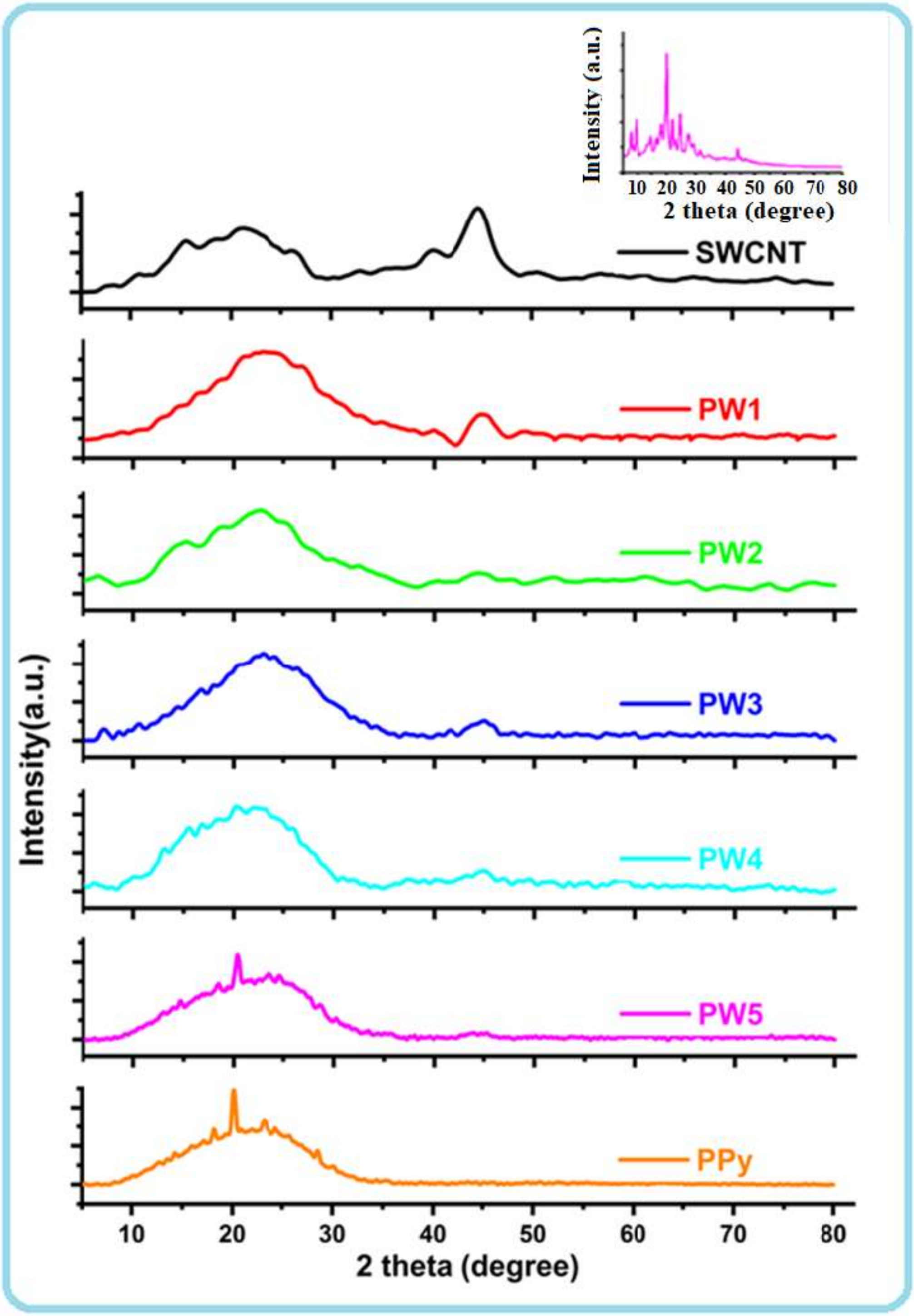

Figure S3. XRD pattern for SWCNT/PPy core shell nanocomposites. XRD patterns of pure SWCNT and PPy are also shown. Spectra of pure SWCNT and PPy are also shown. The inset shows XRD of MO powder.

4. XPS analysis

Figure S4 depicts the XPS survey profiles of pure SWCNT, PPy, and SWCNT/PPy core shell nanocomposites with different PPy layer thicknesses. The C1s of the neat SWCNT is the mean band with no appearance of any other elements, while the spectrum of PPy shows the same band in addition to smaller once of N1s, S2s and S2p. The obtained survey scan for both neat SWCNT [34] and PPy [25] were similar to those values reported in the literature. As for SWCNT/PPy nanocomposites, the intensity of the O1s peak increases marginally with an increase in the coating thickness. The deconvolution of C1s peaks in the XPS spectra of PPy as well as the uncoated and coated SWCNT provide three main components with different intensities as shown in Figure S5 (a–g). The C1s bands of the pristine SWCNT can be observed at 284.94, 286.53 and 290.39 eV corresponding to the C=C, C–O, and C=O bonds, respectively (Figure S5a). As shown in Figure S5g, PPy expresses peaks with binding energies situated at 284.58, 286.15 and 288.39 eV, respectively, which are attributed to C=C, C–N$^+$ and C=O or C=N. The spectra of SWCNT/PPy core shell nanocomposites are almost similar to those of uncoated SWCNT and pure PPy as shown in Figure S4. Peak positions show no notable changes indicating that the SWCNT have been effectively coated by PPy with no extra bonds or crosslinking in the contact surfaces. The perfect π–π stacking between the PPy chains and the SWCNT might also be possible to cause the stabilization of the binding energies of the elements included in both PPy and SWCNT.

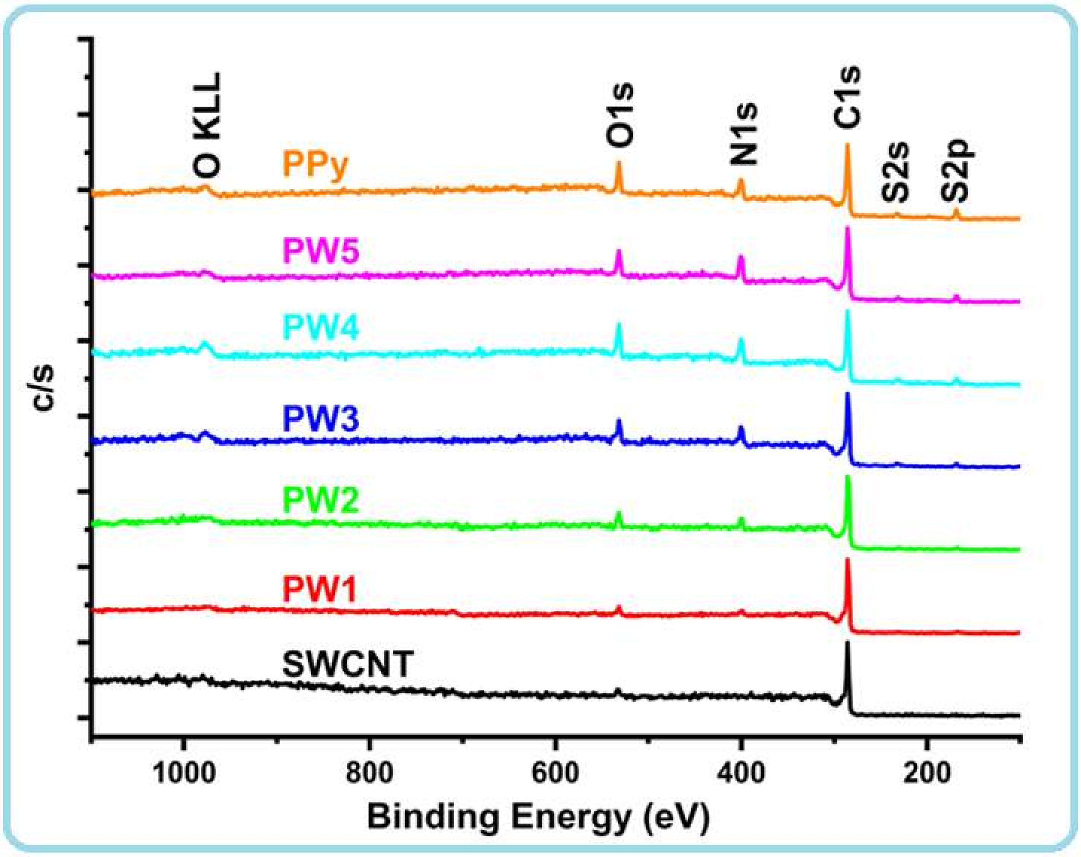

Figure S4. Typical XPS survey profiles of SWCNT, PPy, and SWCNT/ PPy core shell nanocomposites. Profiles of pure SWCNT and PPy are also shown.

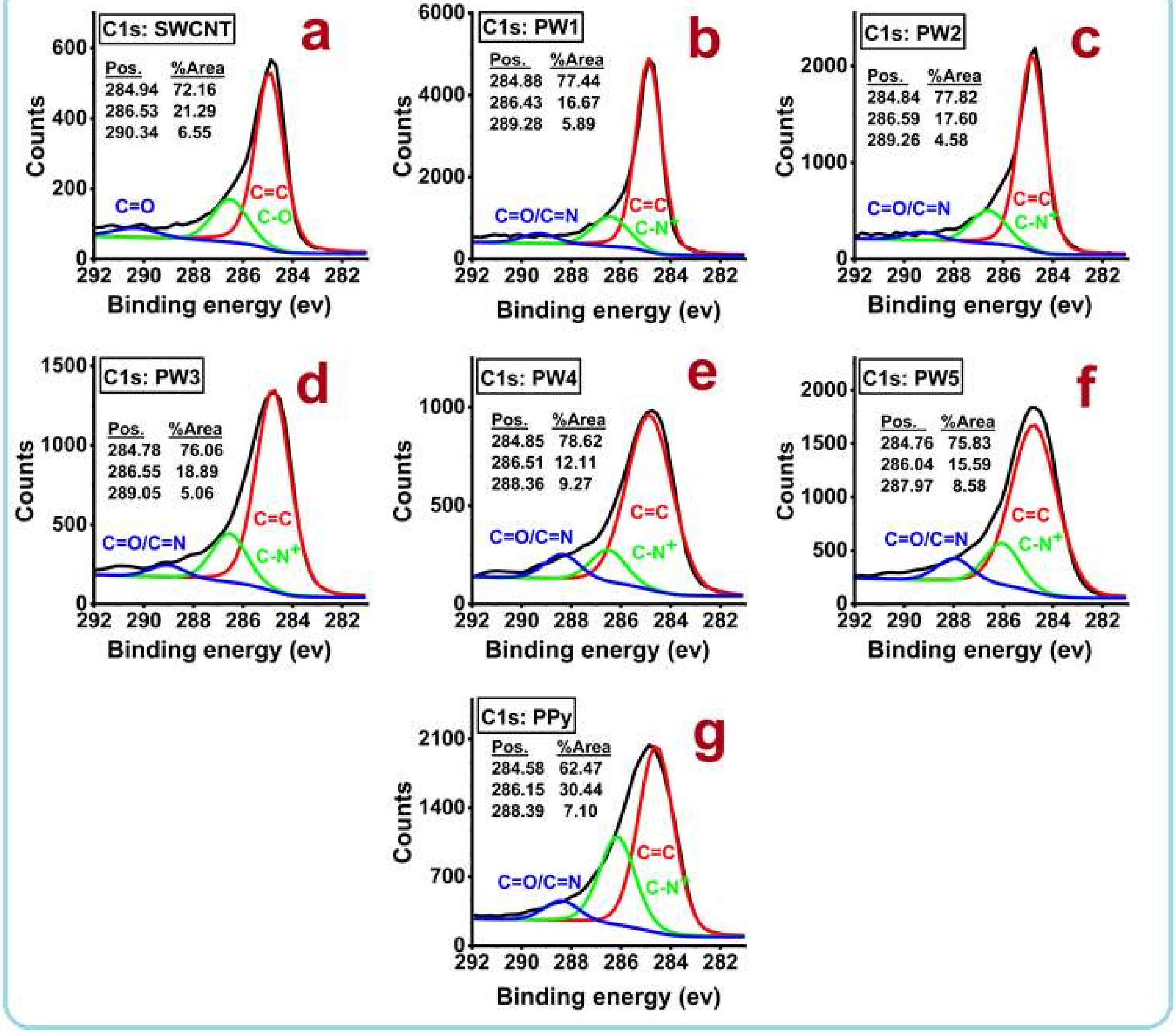

Figure S5 C1s XPS profiles of SWCNT, PPy, and SWCNT/ PPy core shell nanocomposites. (**a**) SWCNT, (**b**) PW1, (**c**) PW2, (**d**) PW3, (**e**) PW4, (**f**) PW5, and (**g**) PPy.

5. ANSYS numerical model

To describe the temperature gradient and electric variation through TE leg module, two main equations, heat flow equation (Equation S1) and continuity equation (Equation S2) are used into energy flow, which can be expressed as follows:

$$\rho c \frac{\partial T}{\partial t} + \nabla q = \dot{q} \tag{S1}$$

$$\nabla\left(J + [\varepsilon]\frac{\partial E}{\partial t}\right) = 0 \tag{S2}$$

where, ρ, c, $\dot{q}$ and ε are the density, specific heat, heat flux and the dielectric permittivity matrix, respectively. The above equations can also be linked by the following equations:

$$q = -[\,k\,].\nabla T \tag{S3}$$

$$J = [\,\sigma\,]\,.\,(\,E - [\alpha].\nabla T\,) \tag{S4}$$

$$E = -\nabla\varphi \tag{S5}$$

where, k, σ and α represent thermal conductivity, electrical conductivity, and Seebeck coefficient matrices for TE leg module, respectively, while symbol φ represents electric po-

tential. In order to solve for temperature (*T*), an electric potential (φ) and current (*I*), equations S3 to S5 can be substitute into equation 1 and 2. The numerical model using ANSYS Mechanical (APDL) finite element software can be used to solve the above governing equations [48–50]. The structural, thermal, and electrical elements can be coupled using 3-D 20-node hexahedron SOLID226 coupled field element. TE material properties, boundary conditions (hot and cold side temperatures), and the geometry of TE module are specified and input into the numerical model [51]. The power output of TE leg is calculated using current and the external resistance (R_0) according to the following power equation:

$$P = I^2 R_0 \tag{S6}$$
